# Scotch Physicians and Surgeons

**Published:** 1888-02-11

**Authors:** C. G. Furley


### Scotch Physicians and Surgeons.

By C. G. Furley.

(Continued from p. 317.)

SIR JAMES Y. SIMPSON (*concluded*).

Although the introduction of chloroform was Simpson's greatest work, the one with which his name is most closely and permanently associated, it was not his only contribution to medical science. He was not the man to sit down quietly before he was forty and say, "I have finished my work." Amidst the care and anxiety of attending to the increasing crowds of patients that besieged him; of giving a large class instruction, both theoretical and practical, in a subject so important as gynæcology; of training nurses to a more thorough and intelligent knowledge of their work than midwives had hitherto possessed; of devising cures for those recondite diseases of women which had till his time defied the skill of the best physicians; amidst, too, the manifold duties and hospitalities which his distinction forced on him—the lion being obliged to stand on his hind paws and dance for the public, as Sir Walter Scott put it—amidst a hundred occupations, professional and social, he found time to carry out new investigations and publish new works.

First came a book protesting against the homœopathic craze, which was then exciting the public; then came a suggestion that consumption might be prevented, or even cured, by the body being regularly rubbed with oil; then an examination into the causes of Asiatic cholera, when that terrible disease was devastating the country; finally, an energetic recommendation of acupressure — the use of metallic ligatures in fastening severed arteries, instead of the silk and catgut ones generally in use. All these works prove the far-reaching intelligence and energy of the man, but though of various shades of value, they have not added in any special manner to his fame. They are the accidents—the by-products of his genius. The same may be said of those antiquarian researches which formed his chief amusement, though these, of course, cannot compare, for interest or value, with even his minor contributions to medicine; but all serve to elucidate the character of a man to whom idleness was the one thing impossible.

Honours and troubles came together in the last years of his life. In 1856 the French Academy of Sciences awarded him the "Monthyon" prize of 2,000 francs, accompanied with a gold medal, for "most important benefits done to humanity." In January, 1866 he was offered a baronetcy by Earl Russell, then Prime Minister. At first he hesitated, because, having not long before lost a considerable amount of money in some ill-advised speculations, he feared that he could not leave his son a fortune sufficient to keep up the position the title would entail; but finally he decided to accept the proffered honour. In June of the same year the University of Oxford conferred on him the degree of D.C.L. In 1868, after he had delivered an earnest and eloquent graduation address to the newly-qualified M.B.s, the municipality of Edinburgh conferred on him the freedom of the city—a civic honour which Scotchmen value highly. This concludes the list of public acknowledgments of his services, unless we include among these the unsuccessful attempt made to induce him to leave his beloved Edinburgh and become a lecturer at King's College, London, and that testimony to the talents of "Sir Simpson," which it was his fate to overhear in a Paris lecture-room.

Against these honours must be set—to take the lesser troubles first—the serious pecuniary losses he met with in the course of several investments, and his failure to obtain the Principalship of Edinburgh University when the death of Sir David Brewster left it vacant. The late Sir Alexander Grant was then preferred to both Simpson and Christison—why, no man knoweth.

But these were not the great sorrows of Simpson's life; his true griefs were the deaths of his son David and his daughter Jessie, whom he was wont to nickname "Sunbeam." David Simpson, who had followed the medical profession, was his father's assistant at the time of his death, and was regarded as his natural successor; and though these posts were creditably filled by Sir James's nephew, who now occupies his uncle's chair, the father's heart received an irreparable blow. On such things as these, however, it is not permitted to the biographer of a mere professional career to do more than touch.

Neither domestic sorrows nor professional disappointments had power to quench his untiring industry; but in the beginning of the year 1870 he began to complain that he was "easily knocked up." In spite of these forewarnings of ill-health he went about as usual, taking long, fatiguing, and hurried journeys to patients at a distance; lecturing, visiting, attending to every one of his multifarious duties. On February 25th, however, angina pectoris declared itself, and he was forced to remain in bed. He suffered much from breathlessness and pain, which the opiates his doctors administered only partly relieved; but at last he sank into a painless unconsciousness, in which he passed away on May 6th, 1870. It is very characteristic of him that when he recognised the fatal nature of his illness, he remarked, "How old am I? Fifty-nine! Ah! I should have liked to do more work."

But his work was now—not ended, but capable of being done without his presence. His pupils, of whom I will mention only two, who have well carried out and extended their master's research—Thomas Keith and Matthews Duncan—have gone all over the world to practise the thoughtful and conscientious methods he taught them, and to hand down the tradition of true gynæcology. Of the boon conferred on the world by the introduction of chloroform it is needless to speak. It has become one of the every-day blessings of the human race; and we of a later generation only wonder how our forefathers lived without it.

A grave in Westminster Abbey was offered, but his family decided that he should be buried by his children in that piece of ground which long ago, when his eldest child died at the age of three, he had bought in Warriston Cemetery, on the north side of Edinburgh, saying: "It is for me and mine." Thither his body was taken, escorted by a funeral *cortège* which included representatives of every public body in Edinburgh, while the mourning inhabitants of a city that loved him and was proud of his work crowded to see the last journey of him who had entered into well-earned rest.

